# Task-Shifting Immunization Activities to Community Health Workers: A Mixed-Method Cross-Sectional Study in Sahel Region, Burkina Faso

**DOI:** 10.9745/GHSP-D-23-00044

**Published:** 2023-10-30

**Authors:** Hamed Sidwaya Ouédraogo, Yewayan Larba Berenger Kabore, Abdoul Ganiyi Sawadogo, Moussa Bakouan, Noaga Sawadogo, Moumai Mano, Amadou Zongo, Simon Sanou, Lassané Kaboré

**Affiliations:** a Sahel Regional Health Directorate, Ministry of Health and Public Hygiene, Dori, Burkina Faso.; b Department of Public Health, Joseph Ki-Zerbo University, Ouagadougou, Burkina Faso.; c AFENET, Ouagadougou, Burkina Faso.; d Terre Des Hommes, Ouagadougou, Burkina Faso.; e National Health Surveillance Service, Ministry of Health and Public Hygiene, Ouagadougou, Burkina Faso.; f National Immunization Programme, Ministry of Health and Public Hygiene, Ouagadougou, Burkina Faso.; g National Health Emergency Response Operations Center, Ministry of Health and Public Hygiene, Ouagadougou, Burkina Faso.; h PATH, Dakar, Senegal.

## Abstract

**Introduction:**

Faced with the frequent disruptions to the health care system and provision of immunization services caused by terrorist attacks that began in 2015, the Sahel region in Burkina Faso initiated resilience strategies, including the task-shifting of immunization activities to community health workers (CHWs). This strategy was designed to involve more CHWs in the vaccination delivery process and ultimately to improve the performance of the health care system.

**Strategy Development and Implementation:**

The task-shifting strategy began as a pilot in Djibo health district in 2019 and then extended to all 4 districts of the Sahel region. CHWs included both personnel recruited through the Ministry of Health and Public Hygiene processes at the national level and other community members who support the operation of health facilities. They were trained on standardized immunization modules and provided with vaccines by functional health facilities teams. Implementation initiated with the administration of oral antigens by CHWs. Subsequently, their service delivery was expanded to include injectable vaccines in the context of the worsening terrorist attacks and the urgent need to protect the health of local populations affected by the security and humanitarian crisis.

**Strategy Evaluation:**

The intervention was evaluated through an internal programmatic review conducted as a descriptive cross-sectional study implemented from August 1 to October 28, 2022, in the Sahel region, including a survey in Dori health district. CHWs involved in implementing the strategy were considered for interviews. Performance indicators for all antigens have shown an upward trend since the strategy's inception in 2019.

**Conclusion:**

The task-shifting of immunization activities to CHWs has been implemented successfully in a region seriously affected by terrorism-related insecurity. It holds the promise of maintaining or even improving performance if institutionalized and scaled up while improving the monitoring of adverse events following immunization by the CHWs.

## Abstract (precis)

The authors describe a successful strategy of task-shifting child immunization activities to CHWs in insecure areas of Burkina Faso to improve vaccine coverage.

## Teaser

**Key Findings:**

- As part of Burkina Faso's national health system resilience plan, a task-shifting strategy was piloted in 1 district using community health workers (CHWs) to provide child immunization services to hard-to-reach populations in areas affected by terrorist attacks.
- Vaccine coverage improved for all antigens administered.
- Essential factors of the strategy's success included capacity-building of CHWs, adequate planning to ensure a vaccine supply, communication activities to increase community awareness and demand, and routine data collection and monitoring.
- After the experience in the Sahel region, the strategy was scaled up in the whole Sahel region and integrated into national guidelines.

**Key Implications:**

- National immunization guidelines should be adapted and used by governments and implementing partners to strengthen the credibility of a task-shifting strategy.
- Task-shifting strategies must take into account the CHWs' training, supervision, and remuneration, as well as additional financial costs that CHWs incur in performing their duties.
- We call on health system managers to build strong community health systems so countries or regions are better prepared to withstand crises and shocks.

## INTRODUCTION

Improving maternal and child health is a key objective of health care systems worldwide. To achieve this, a number of public health interventions are being implemented to reduce the burden of childhood morbidity and mortality, which has previously been relatively high in sub-Saharan Africa.^1^ Vaccination has significantly reduced the number of deaths from infectious diseases.

Vaccines also reduce disability.^2^ The antigens and procedures used in immunization are subject to rigorous quality standards, which must be maintained in all contexts.^2^^,^^3^ In Burkina Faso, the child immunization program has been implemented consistently since 1980 by health facilities through routine delivery or several supplementary immunization activities.^4^

However, since the beginning of terrorist attacks by armed groups in 2015, the health care system has been frequently disrupted, including the provision of immunization services. In 2021, the Sahel region had an estimated population of 1,526,160.^5^ The demographics of the region have been significantly altered by the security crisis and was home to more than 497,248 internally displaced persons at the end of December 2022.^6^

The health care system is structured around 3 levels. At the peripheral level, the first point of contact with health services is the health facility.^7^ The Sahel region theoretically had 134 health facilities on December 31, 2021, of which 71 (53%) were closed due to insecurity on December 27, 2021 (compared with 56 as of December 30, 2019).^8^ The humanitarian crisis has exacerbated the problem of equity that already existed in the region, where average distances to a health facility were already very high, making access difficult for communities. The disruption of health services has led to low population coverage of essential health services, including vaccination. For example, in the Sahel region of Burkina Faso (among the hardest hit by terrorist attacks), coverage for the third dose of the diphtheria, tetanus toxoid, and pertussis vaccine was 51% in 2020, compared to a national coverage of 99%, as a result of the closure of a large number of health facilities.^9^^,^^10^

Yet, vaccines are essential for the prevention and control of many communicable diseases, making them a pillar of global health security.^2^ Burkina Faso is currently developing its national immunization strategy based on the 2030 Immunization Agenda, which calls for better coverage of hard-to-reach populations and a strengthening of all actions to improve demand for immunization services.^2^

Various initiatives have been launched as part of Burkina Faso's 2019 health system resilience strategy, including the development of vaccination strategies in insecure areas.^11^^,^^12^ This led to the decision to implement the task-shifting of immunization services to community health workers (CHWs) in the Sahel region. CHWs are community members with a primary school certificate who are selected through a community-led process and trained on a series of activities conducted within the communities where they live. They receive a monthly incentive of US$34 per month from the government and its partners, including the Global Fund.^13^

The development of vaccination strategies in insecure areas is part of Burkina Faso's health system resilience strategy.

In this article, we describe the task-shifting strategy designed and implemented in the Sahel region of Burkina Faso in terms of content, implementation process, and results achieved.

## TASK-SHIFTING STRATEGY DESIGN AND IMPLEMENTATION

### Strategy Planning

**FIGURE 1:**
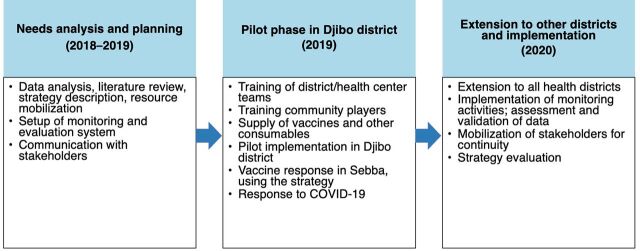
Key Development Dates During the Pilot Phase of Task-Shifting Immunization Activities to Community Health Workers, Sahel Region, Burkina Faso

After the health facilities performance analysis, the Sahel regional health directorate team designed the strategy in collaboration with the health district management teams. However, before this, consultations were held with national bodies, including the national directorate in charge of prevention through vaccinations and international partners, such as the country office of the World Health Organization. The strategy planning required prioritization of health facilities based on financial resources available for CHW training and the feasibility of CHWs to commute commuting for the distribution of supplies and vaccination in villages. As part of the development of the implementation plan, the Sahel Regional Health Department team estimated the target area to be covered. It then estimated the number of doses of vaccine needed and the budget required. To mobilize the financial resources needed for the training courses, the team held meetings with its partners and identified government resources at the district level that could be used. The main stages of implementation are shown in Figure 1.

### Strategy Piloting

The selection of health facilities for implementation was a 2-step process (Table 1).

1. Assessment of the recurrence and risk of terrorist attacks by attempting to count and map them by health care facility.
2. Assessment of the vaccination performance of health facilities using measles-rubella (MR) vaccination coverage over the last 2 years (2017 and 2018).

**TABLE 1.** Vaccination Coverage Rate for First and Second Doses of Measles Rubella Vaccine by Health Center, Djibo District, Burkina Faso^a^

	Measles Rubella Vaccine			
	First Dose, %		Second Dose, %	
Health Centers	2017	2018	2017	2018
1. Badnogo	95	88	71	59
2. Baraboulé	95	66	65	39
3. Bossey	98	65	100	47
4. Bouro	93	84	51	43
5. Béléhédé	121	80	91	52
6. Damba	89	5	72	5
7. Diguel	105	115	91	69
8. Djao-Djao	64	92	56	50
9. Filio	86	88	80	68
10. Gaik-Goita	134	109	131	128
11. Gasseliki	97	100	67	88
12. Gasselté Paoua	100	103	82	91
13. Gomdé	98	34	91	25
14. Kourfadji	76	ND	26	ND
15. Koutougou	113	28	63	27
16. Kouyé	N/A^b^	57	N/A^b^	N/A^b^
17. Nassoumbou	104	79	97	68
18. Petega	44	28	10	21
19. Pougzaibaogo	108	130	77	281
20. Pétégoli	89	73	61	63
21. Sikiré	113	102	78	80
22. Sona	97	93	118	113
23. Soumbella	10	N/A^b^	N/A^b^	N/A^b^
24. Yalanga	94	58	70	N/A^b^
25. Belahouro	N/A^b^	N/A^b^	N/A^b^	N/A^b^
26. Liki	N/A^b^	N/A^b^	N/A^b^	N/A^b^
27. Sibbé	N/A^b^	N/A^b^	N/A^b^	N/A^b^

Abbreviations: N/A, not available; ND, no data.

a Source: Office in charge of health statistics and epidemiological surveillance of the Sahel regional health directorate, 2018.

b Data not available due to an opening in the year concerned or a closure due to the terrorist attacks.

This process led to the selection of the first health facilities (n=27) in Djibo health district based on MR1 and MR2 vaccination coverage in 2017 and 2018.^10^ In the first phase, we selected health care facilities with low coverage. After the pilot phase, the strategy was taken into account in the annual plans of the 3 other health districts (Gorom Gorom, Sebba, and Dori). The extension was completed in 2020, covering all 4 health districts in the Sahel region of Burkina Faso.

### Capacity-Building

The strategy relied on CHWs living in the intervention zones, who were recruited and incentivized (US$34/month) by the Ministry of Health in 2016.^13^^,^^14^ District teams trained the head nurses and immunization officers of the selected health facilities, who in turn trained the CHWs for 7 days at the site. Training at the health facility focused on immunization basics (including the reasons behind the creation of the national immunization program) and the technical aspects of immunization (with a focus on the vaccine, cold chain, administration techniques, and reporting). Participants then attended a 4-day practical phase that consisted of assisting health staff within the health facility (passive observation) and practicing immunization delivery tasks under the supervision of health staff responsible for the validation of CHW skills. Task-shifting was first carried out with oral antigens (oral polio vaccine and rotavirus vaccine), followed by the administration of injectable antigens, which were first handled by CHWs during the response to a serogroup C meningococcal meningitis outbreak in the Sebba health district in 2019.^15^ During the training, injectables were first practiced on local materials, such as mattress covers, before moving on to vaccination of children. CHWs were evaluated during this practical phase, and their skills were validated. In general, those who completed this training process had very high practical skills and a reassuring ability to vaccinate. Supervision of these CHWs trained on vaccination has continued beyond their initial training as part of the monthly monitoring activities conducted by the health facility teams.

### Supply Chain

To ensure sustainability, vaccine supply was designed to use the health system operations with adaptations. CHWs obtained supplies from the nearest health facilities. To do this, health facilities had to provide CHWs with vaccine carriers (or isothermal crates for remote villages). These are then transported to the point of service delivery by cart, motorcycle, or bus. CHWs living in places where health facilities are not functional could use the refrigerators of these health facilities with the support of district immunization focal points for maintenance. In the absence of functioning refrigerators, CHWs traveled between the villages and the nearest functional health facility and received their supplies in vaccine carriers according to their needs at vaccination sites. The planning of vaccination sessions and the supplying of vaccines were done according to a schedule defined by health facility teams, and CHWs' health facilities were responsible for rationing the doses to limit wastage. The rest of the supply chain, from the regional vaccine store to districts, remained unchanged.

### Communication

Communication was an important dimension in the implementation of the strategy. It was addressed through direct engagement with the various communities, advocacy with local authorities, home visits, and the use of town criers to convey messages, where possible, during mass campaigns (at regional, district, or local levels).

Communication was an important dimension in the implementation of the strategy and was used to address the population's doubts and prevent poor acceptance of vaccination.

These communication activities aimed to address the population's doubts and prevent poor acceptance of vaccination by the community, particularly regarding the administration of injectable products by CHWs. The CHWs used educational talks and home visits to raise community awareness. Emphasis was placed on CHWs' ability to communicate the benefits of immunization as outlined in the child health promotion leaflets. Vaccination calendar posters were also distributed to CHWs.

Communication about scheduled vaccination sessions was made door-to-door by the CHWs on the eve of the activity to get parents involved. The tools used were those recommended by Burkina Faso's Expanded Programme on Immunization, based on the WHO guide^16^ and contained in the various guides and directives developed as communication materials. The image boxes and posters developed with the advent of COVID-19 provided additional resources for CHWs to communicate with communities. Specific key messages emphasized by the CHWs included information about the diseases against which vaccines protect and the life-saving benefits for children, as well as broader benefits of immunization, including the time and money saved if vaccinated persons are healthy enough to take care of their hard-won assets such as livestock.

### Biomedical Waste Management

CHWs were required to ensure proper cleaning before and after vaccination sessions by collecting biomedical waste produced during the session. CHWs were trained according to the national vaccination guide,^16^ which indicates how vaccination sites operate and how biomedical waste should be managed. The waste generated in the villages had to be transported by CHWs to health facilities for disposal. CHWs were instructed to collect sharps (syringes) in safety boxes (before transportation to health facilities), and other types of waste (including packaging) were conveyed and destroyed at the health facilities. Health facilities must destroy all biomedical waste with their own incinerators or convey needles and other sharp instruments to national centers during post-campaign opportunistic biomedical waste collections.

### Data Management

Activities were conducted under the coordination and supervision of the health facility teams. When a health facility was closed, responsibility for coordination fell to the nearest health facility, assisted by the district immunization focal point. The standard health information system data collection forms were reproduced and used.^17^ After vaccination, CHWs transmitted the data as soon as they had an opportunity to visit the health facility supplying the vaccines, most often by bus. This was usually done when they traveled to get vaccines for vaccination sessions. Data could also be sent by manual letter to anyone visiting the health facility. Opportunities to send supplies to CHWs were used by the health facility manager to discuss any data reporting issues. These data were then forwarded to the person in charge of epidemiological surveillance and the health district information system. An effort was made to comply with the national health information system transmission levels and tools. Data were then entered into the health facilities' monthly activity reports and finally into the DHIS2 at the national level.

## STRATEGY EVALUATION METHODOLOGY

The aim of this evaluation was to assess the functionality of immunization service delivery by CHWs after 2 years of implementation. More specifically, we sought to identify lessons that could improve and strengthen this task-shifting strategy for immunization services in such a complex and changing environment.

In our evaluation, we sought to identify lessons that could improve and strengthen the task-shifting strategy in this complex and changing environment.

We carried out a process evaluation based on the requirements described by Ridde in *Evaluation Practices and Methods in Africa*.^18^ The process elements and immediate results were assessed to provide a value judgment to guide any other health system wishing to implement this strategy.

### Evaluation Design

Our evaluation focused on the implementation process. To gain a broad understanding of the lessons learned from this vaccination task-shifting strategy, we opted to carry out a mixed-method evaluation combining quantitative and qualitative methodologies. We used data generated by health services for activities between 2016 and 2022 (all inclusive) extracted from DHIS2 and data from other sources at the level of the Sahel regional health directorate. Primary data collection (quantitative and qualitative) took place between August 1 and October 28, 2022, in the Sahel region of Burkina Faso.

### Target Population

Our evaluation focused on children aged 0–59 months, conventional health workers, and CHWs in the evaluation area.

### Sampling

Quantitative data were collected from CHWs according to geographical distribution and whether they were involved in the implementation of the strategy in Dori health district, where the regional health directorate is headquartered. This convenience sampling of respondents (CHWs) was essentially driven by security reasons and the availability of financial resources to collect data. The selected health facilities were Lamdamol, Selbo, Gotougou, Katchirga, and Sampelga—all rural facilities in Dori health district—with a sample of 31 CHWs.

Qualitative data was collected remotely by telephone, involving 28 CHWs implementing vaccination activities in the Dori, Gorom Gorom, and Sebba health districts. The region's fourth health district, Djibo, was deprived of a functioning telephone network for its rural health facilities due to the sabotage of telecommunication equipment by armed groups and was therefore not included in this evaluation. In addition, an exhaustive list of children aged 0–59 months vaccinated between 2017 and 2021 and recorded in the DHIS2 database was used.

### Data Collection Tools and Analysis

Both quantitative and qualitative data collection techniques were used for data collection. For the quantitative component, we designed a document review grid that enabled us to capture data extracted from the national data management portal (DHIS2).^19^ Additional quantitative data collected from CHWs in the Dori health district were collected using a survey questionnaire. For the qualitative data collected from CHWs in Dori, Gorom Gorom, and Sebba, a semidirective interview grid was used, both remotely and by telephone. Data collection tools captured information about 3 groups of variables.

- Demographic characteristics: occupation, place of residence, level of education, distance between the village and the health facility site, length of service, etc.
- Operations: training, effectiveness of interventions, existence of financial support, nature of district support, community support, availability of equipment, type of antigen administered, supply method, etc.
- Vaccine coverage: bacille Calmette-Guérin, oral polio vaccine 1 and oral polio vaccine 3, Penta1 and Penta3, MR1 and MR2, and meningococcal A coverage rates.

Quantitative data were then entered into Excel and analyzed using Excel and SPSS 22. Qualitative data from CHW interviews were recorded and transcribed into MS Word in French, the language used by these CHWs, and analyzed after verbatim coding using MAXQDA software.

### Ethical Approval

We obtained administrative authorization from the Ministry of Health. Before the start of data collection, we also obtained administrative authorization from the Sahel Regional Health Department, district medical officers, and municipalities. Data were stored on electronic media accessible only to the restricted evaluation team and protected by an access code. As this work was considered to be an evidence-based program evaluation, it was not submitted to the national ethics committee for further review.

## RESULTS AND BENEFITS

### Sociodemographic Characteristics of Sampled CHWs

The quantitative survey included 31 CHWs in Dori health district who consented to participate. As shown in Table 2, 74.2% (23) of them were male. Among participants, 41.9% (13) had neither motorcycles nor bicycles (Table 2). The majority were farmers by profession, with 77.4% (24) having at least primary school education (Table 2). They all confirmed that they had implemented task-shifting after receiving training.

**TABLE 2.** Sociodemographic Characteristics of Community Health Worker Respondents in the Task-Shifting Survey, Dori Health District, Burkina Faso

	Male, No. (%)	Female, No. (%)
Gender	23 (74.2)	8 (25.8)
Means of travel		
Foot	11 (47.8)	2 (25.0)
Bike	1 (4.4)	2 (25.0)
Motorbike	11 (47.8)	4 (50.0)
Education level		
None	1 (4.4)	0 (0)
Literate	4 (17.4)	2 (25.0)
Primary	9 (39.1)	4 (50.0)
Secondary	9 (39.1)	2 (25.0)
Profession		
Farmer	29 (93.5)	
Trader	1 (3.2)	
Student	1 (3.2)	

The qualitative interview by telephone reached 32 CHWs working in villages and linked to 24 health facilities. The average median age of participants was 31.32 years (standard deviation ±8.13; range: 20–52).

### Effectiveness of Task-Shifting and Health Facility Performance

The administration of injectable vaccines by CHWs began in December 2019, with the supervision of 40 CHWs trained initially in the town of Djibo and providing vaccinations in areas where internally displaced persons gathered. The strategy was subsequently extended to other districts. By the end of 2021, the region had 332 CHWs trained in the strategy and regularly providing vaccination in their localities.

**FIGURE 2:**
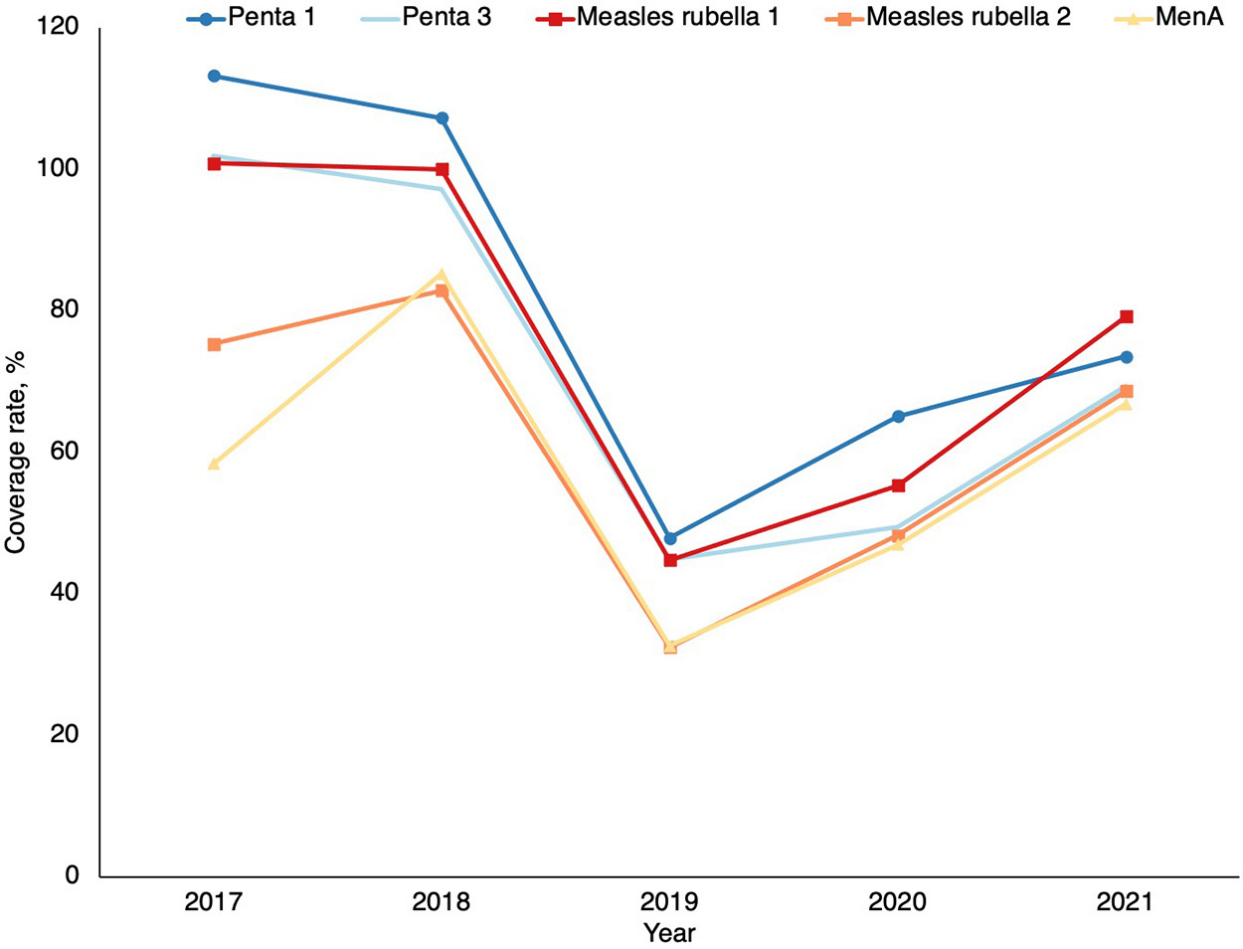
Evolution of Vaccination Coverage of the Sahel Health Region, Burkina Faso, 2017–2021 Abbreviations: measles rubella 1, first dose of measles rubella vaccine; measles rubella 2, second dose of measles rubella vaccine; MenA, meningococcal type A vaccine; penta 1, first dose of pentavalent vaccine; penta 3, third dose of pentavalent.

**FIGURE 3:**
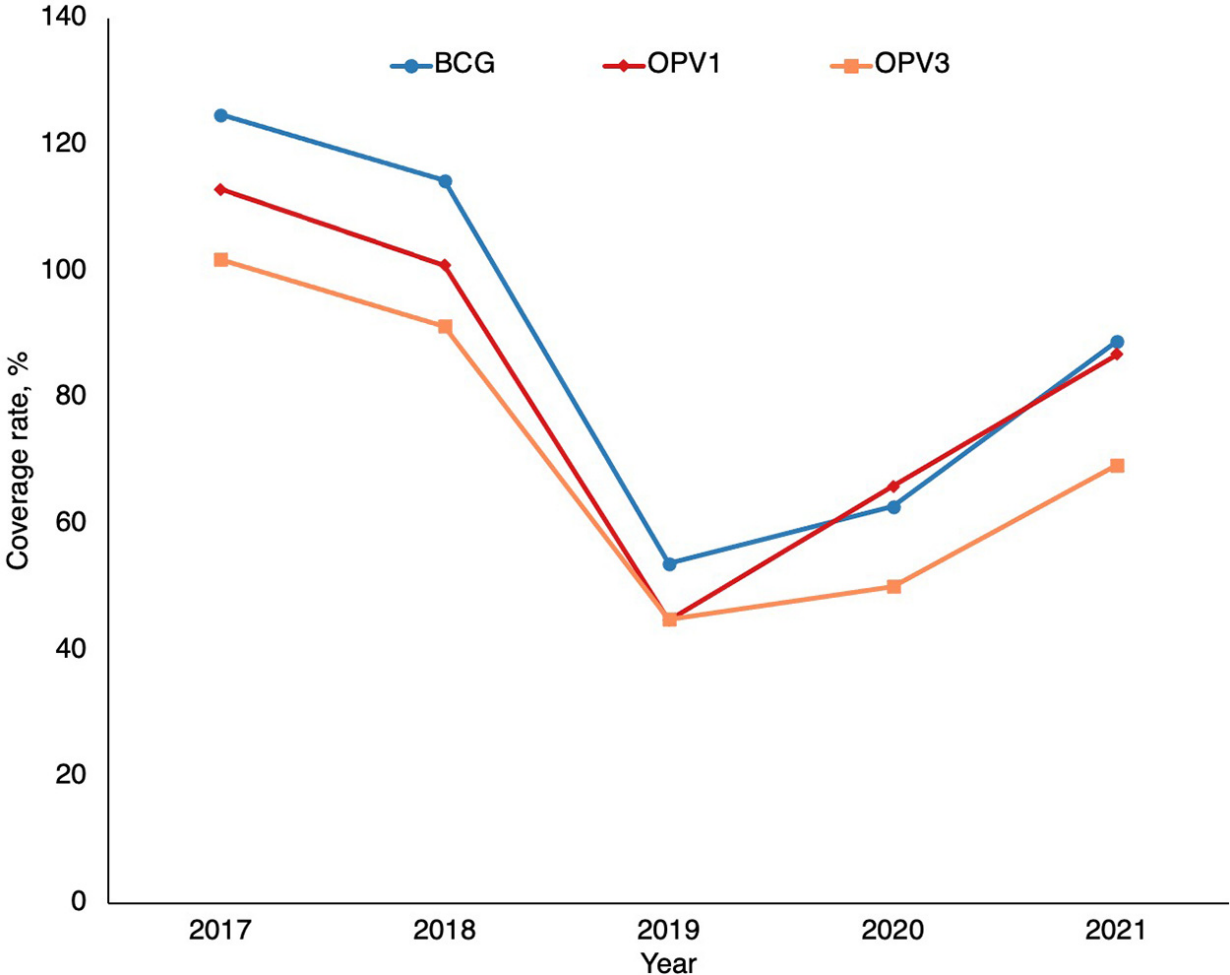
Evolution of BCG, OPV1, and OPV3 Vaccination Coverage From 2017 to 2021 in the Sahel Region of Burkina Faso Abbreviations: BCG, bacille Calmette-Guérin vaccine; OPV1, first dose of oral polio vaccine; OPV3, third dose of oral polio vaccine.

The task-shifting to CHWs enabled 6,223 children to be vaccinated, including 180 in their first contact with vaccination services, according to data from the statistics office of the Sahel regional health directorate. The resulting data, available in DHIS2, show that the strategy has improved vaccine coverage performance in the Sahel region (Figure 2 and Figure 3). Overall, there was a downward trend in performance from 2017 to 2019 and an upward trend from 2020 onward during the strategy implementation period.

Data indicate that the strategy has improved vaccine coverage in the Sahel region, reversing a downward trend before the implementation period.

The emergence of COVID-19 led to a number of restrictive measures that had an impact on vaccination services. The use of CHWs enabled vaccination to continue with smaller numbers of people, often door-to-door, and to comply with the social distancing measures recommended at the height of the pandemic.

### Biomedical Waste Management

The reports and results obtained indicate that the application of biomedical waste management guidelines has been effective from the outset. The survey of the Dori district showed that 64.5% of the CHWs surveyed (n=31) stated that they bring their vaccine waste to the health facilities for incineration. According to the same survey, some CHWs bury and burn their waste.

### Additional Financial Costs

In the implementation of this activity, the majority of CHWs were recruited by the government of Burkina Faso, which provides them with a monthly incentive of US$34. A few were volunteers at the health facilities, usually members of management committees or helpers managing the medicine store.

However, a number of additional costs were reported in the implementation of daily activities linked to the distance traveled (Table 3) and means of transport (Table 2). These costs are significant in areas where the number of children is high, as CHWs are obliged to make more frequent trips from the village to the functional health facilities for supplies.

**TABLE 3.** Distance Traveled by Community Health Workers From Village to Health Center, Dori Health District, Burkina Faso

Distance, km	No. (%)
0	8 (25.8)
5	7 (22.6)
6	1 (3.2)
8	5 (16.1)
15	1 (3.2)
18	2 (6.5)
20	2 (6.5)
25	1 (3.2)
27	3 (9.7)
45	1 (3.2)
Total	31 (100.0)

### Difficulties Identified

The main difficulties identified during interviews with CHWs related to issues such as the unavailability of a suitable means of transport, such as motorcycles, or related logistical challenges. The CHWs that did not have a motorcycle or bicycle made every effort to carry out their activities either by borrowing a means of transport, using vehicles from which to reach village markets when available, or using carts.

*I walk 7 km to get to the vaccination site. I don't have a motorcycle and the nurse joins me with the vaccines.* —CHW

*The health facility motorcycle is very old, it needs a lot of repairs: they say it's up to the Town Hall to reimburse me or do it, but so far nothing concrete.* —CHW

*I miss my program (or I arrive late because I'm on foot), because I don't have a motorcycle.* —CHW

Respondents also mentioned a lack of means to finance the cost of the fuel needed for these transports.

*Yes, there's a problem: I have my own motorcycle, but sometimes I walk to vaccinate because there's no fuel or money for fuel.* —CHW

*[I] take a motorcycle cab at a cost of 500 FCFA to be paid in 2 installments in order to collect the vaccine or to work at the vaccination site every Thursday (vaccination day). No one has ever given me anything.* —CHW

These expenses are incurred despite their criticism of the amount of monthly allowance provided by the government.

*I receive 20,000 FCFA, which is not enough for fathers of families, and it takes a long time to come, you have to credit all the time, and it doesn't look good: they don't even trust us anymore.* —CHW

*The incentives (the 20,000 FCFA) haven't arrived for 10 months; it demotivates us, and we're forced to look elsewhere, including the gold mines in Côte d'Ivoire.* —CHW

Finally, our analyses allowed us to perceive the presence of risk and fear in the comments of these community members.

*We take a lot of risks, to save the children.* —CHW

Another CHW reported the presence of armed men in his area of operation.

*I've already come across them 4 times. Thank God, I've only been bothered once. People say I'm crazy, but I'm not. It's a sacrifice; my work as a CHW.* —CHW

## DISCUSSION

The task-shifting strategy was initially inspired by the security context and then became critical in the resumption of immunization services after the interruption caused by COVID-19.^27^ The task-shifting of vaccination activities to CHWs in the Sahel region has enabled the continued provision of vaccination services to hard-to-reach populations that are undergoing a scourge of insecurity—a level of risk confirmed by the assertions of the CHWs interviewed who occasionally encountered armed men during vaccination activities. These CHWs have already played an important role in the provision of health care in Burkina Faso and in several other countries.^13^^,^^20^ The implementation of this strategy has made it possible to maintain health protection even amid the closure of health facilities or an upsurge in terrorist attacks. CHWs justify the implementation of these activities in a crisis context by a desire to help their communities, according to CHWs' responses.

The implementation of the task-shifting strategy has made it possible to maintain health protection even amid the closure of health facilities or an upsurge in terrorist attacks.

The task-shifting strategy makes it possible to fill major gaps in the health workforce that existed before the COVID-19 pandemic. The World Health Organization estimates the health workforce at 18 million health workers worldwide.^21^ Task-shifting is an approach that helps to ensure fair, equal, and continuous access to health care services, particularly in the event of a major shortage of qualified staff leading to service interruptions. It aligns with previous policies and strategies in Burkina Faso to actively seek all children that are the focus of the Expanded Program on Immunization. The strategy provides communities with better access to immunization services closer to where they live. Bringing health care closer to home and addressing the social determinants of routine immunization partly explain why the coverage achieved by the evaluation was quite high.^16^^,^^22^

Task-shifting vaccination services is an appropriate way to move toward better population access to primary health care.^23^^,^^24^ This approach is fully in line with the community health strategy that the country developed in 2018, as well as with its investment plan.^14^^,^^25^ Although the strategy has improved vaccination coverage, as shown by the trends in Figure 2 and Figure 3, in future studies, it would be suitable to address all the factors and identify those associated with this improved performance using a different analytical design.

Our findings lead us to recommend the implementation of this strategy in areas facing a security crisis or other major crises and to argue for greater involvement of CHWs in health programs and disease management. Burkina Faso has used CHWs in task-shifting for the management of childhood illnesses such as malaria, diarrhea, and pneumonia.^14^ CHWs are also involved in the implementation of self-injection in family planning with good results. Successful experiences of CHW task-shifting have also been reported in several other countries. In Nigeria, after having involved CHWs in maternal and child health programs, management of infectious diseases, and supply of contraceptive implants, some program managers now use informal mechanisms to involve these CHWs in hypertension and diabetes care^26^ to help the country achieve its objectives in the fight against noncommunicable diseases. This shows us the additional opportunities that Burkina Faso can explore in moving toward universal health coverage.

This strategy was of vital importance during the implementation of the COVID-19 immunization roll-out plan in the Sahel region that required substantial effort to overcome reluctance and generate community support, similar to previous experiences with other vaccines, such as the polio vaccine.^23^^,^^28^^,^^29^

The strategy contributes to strengthening equity in immunization by facilitating coverage of the Sahel region, which faces a security crisis involving many internally displaced persons. It has enabled the maintenance of vaccination services despite the risks faced by communities, which would probably not have been borne by health facility teams. Increasing the availability of resources for the strategy's effective and sustainable implementation, including the strengthening of a cold chain more adapted to the context (isotherms, vaccine carriers, and solar refrigerators), could improve vaccine safety.

Interviews with CHWs suggest that the application of selection criteria and refresher training to keep current with directives were essential to the success of the strategy. These CHWs have a generally satisfactory level of education, according to the Dori district survey. The profile of CHWs complies with the national guidelines governing their recruitment in Burkina Faso.^13^ One interviewee mentioned the importance of supervision by the most experienced CHWs assisting the health facility teams even before the onset of the crisis. This peer support was key to resolving some of the difficulties often faced by CHWs that required the experience or input of health training teams. One specific area of effective peer support was in catch-up situations where the child was unable to keep to the vaccination schedule.

Generally, supervision is proving to be a major success factor in initiatives to involve CHWs in service provision. It has been crucial in the integration of community activities during the involvement of adolescents in the provision of adolescent-friendly reproductive health services in Zambia.^20^^,^^30^^,^^31^ The challenge will be to continue this supervision while using interactive means, such as prerecorded videos and audio lessons that can be accompanied to correct any shortcomings. Although additional financial support will be needed for these resources, it will likely be less than that required by mobile initiatives, such as mobile clinics that mobilize health workers over long distances and require substantial human and material resources. Health economics studies would make it possible to estimate the lives saved and the impact of the strategy on health care expenditure.

The interviews conducted enabled us to note additional financial needs, with a suggestion to increase the amount of financial incentives given by the government to CHWs and to set up a clear mechanism at the health district level to support the cost of fuel that some CHWs are required to bear on their own. CHWs recruited by the Burkina Faso government receive a monthly allowance of around US$34, which is far from enough to cover their related expenses. These related expenses could be borne by direct allocations to CHWs as part of the financing of outreach immunization services. The continuity of the strategy is reassuring, given the government's commitment, in particular, with a state budget line to cover CHWs' monthly allowances and the inclusion of community strategies in the strategic orientations of the national health development plan. However, despite this commitment, material resources should be mobilized through various Ministry of Health projects to facilitate implementation of the strategy by health establishments. The continuation of activities requires better support for the mobility of CHWs (e.g., fuel and support for motorcycles when they can be used). Implementation of the strategy is highly dependent on travel and logistics, and some CHWS have indicated the use of their own motorcycles for service activities.

The continuation of activities requires better support for the mobility of CHWs, as implementation of the strategy is highly dependent on travel and logistics.

The system must also provide more resources to improve the quality of strategy implementation. Indeed, some CHWs had to walk long distances despite the many trips, which could lead to exhaustion and abandonment of the work, yet the strategy calls for the use of small quantities to avoid deteriorating vaccine quality. Activities continued relatively smoothly in localities where solar refrigerators were available, regardless of the functionality of the health facility. In situations where the cold chain was not available, CHWs traveled back and forth to obtain stocks of vaccines and supplies needed for vaccination sessions in their localities. There is a lack of equipment for transporting and storing vaccines. This has led us to recommend the provision of more durable means (refrigerators and vaccine carriers) for solar-powered vaccine storage at the community level.

According to the survey results in the Dori health district, CHWs are making an effort to bring biomedical waste back to health facilities and manage it with incinerators or, in the worst case, burn and bury it. However, our evaluation has revealed a number of difficulties faced by stakeholders in transporting waste to health facilities—the main practice of community workers in vaccine waste management. These difficulties are essentially logistical, namely the lack of financial resources and motorcycles for transporting waste to health facilities for destruction. Vaccine waste management could also be disrupted by shortages of supplies, in particular garbage cans for sharp items such as needles, and difficulties in transporting vaccine waste.

Data were collected using tools from the national health information system. We are able to trace and integrate data into aggregate health data. However, we note that the reporting of adverse events following immunization must be strengthened, given the very low reporting rate since the beginning of the strategy. Monitoring and evaluation with dissemination of reports are essential in this context to document the safety of injectable vaccine administration by CHWs, which could reassure communities and other public health actors who may still have concerns. The provision of immunization services is an international priority and an essential component of children's right to be considered in emergencies.^32^

### Recommendations

Considering the results of this evaluation, we propose the following recommendations.

- Increase the financial motivation of CHWs in the Sahel region and other insecure areas who implement community-based interventions, including immunization.
- Provide health facility teams and CHWs with financial resources for refresher training and supervision.
- Strengthen the cold chain with adequate material and energy autonomy for CHW activities in the Sahel region.
- Provide CHWs with interactive training tools such as audiovisual firmware, audio messages, and pocket memos on immunization for self-training and distance coaching.
- Strengthen the availability of resources for surveillance of adverse events following immunization at the community level.
- Provide health facilities with resources for waste management (e.g., fuel costs for transport, adapted motorcycles, and boxes for needles).
- Strengthen the integration of services at the community level for the delivery of a broader package of services by CHWs.

### Limitations

This evaluation has its limitations, as it did not take into account the overall cost of the strategy in its design, and its inclusion in future evaluations will undoubtedly reinforce the strategy's sustainability. Also, future studies could assess beneficiary satisfaction and use an analytical study design to investigate all factors associated with improved immunization coverage.

## CONCLUSION

Task-shifting of immunization services is being implemented in the Sahel region of Burkina Faso with encouraging results. Analyses of the results obtained between 2017 and 2021 show the positive influence of task-shifting in immunization. This strategy is now institutionally anchored with its adoption in many health system resilience tools, such as vaccination guidelines in security-compromised areas. Task-shifting holds the promise of enabling the continuity of primary health care, including immunization, when conventional health workers can no longer operate due to insecurity. The successful and sustainable implementation of the task-shifting strategy will require a reliable supply of vaccines and related supplies and adequate funding to pay for CHWs and their activities. Finally, periodic evaluations of the strategy will be necessary for ongoing learning, adjustments, and improvements.

## Notes

**Peer Reviewed**

**First published online:** September 28, 2023.
